# Vinorelbine alternating oral and intravenous plus epirubicin in first-line therapy of metastatic breast cancer: results of a multicentre phase II study

**DOI:** 10.1038/sj.bjc.6602588

**Published:** 2005-05-31

**Authors:** D Serin, M Verrill, A Jones, T Delozier, R Coleman, E-D Kreuser, K Mross, B Longerey, M Brandely

**Affiliations:** 1 Institut Sainte Catherine, BP 846, 1750 Chemin du Lavarin, 84082 Avignon Cedex 02, France; 2 Northern Center for Cancer Treatment, Newcastle NE4 6BE, UK; 3 Royal Free Hospital, London NW3 2QG, UK; 4 Centre François Baclesse, Caen 14076, France; 5 Weston Park Hospital, Sheffield S10 2SJ, UK; 6 Krankenhauss der Barmherzigen Bruder, Regensburg 93049, Germany; 7 Klinik Fur Tumorbiologie, Freiburg 78106, Germany; 8 Institut de Recherche Pierre Fabre, Boulogne 92654, France

**Keywords:** metastatic breast cancer, oral vinorelbine, epirubicin, oral chemotherapy

## Abstract

The combination of intravenous (i.v.) vinorelbine and epirubicin is highly active in the treatment of metastatic breast cancer (MBC). In an effort to improve patient convenience, we investigated a regimen alternating i.v. and oral vinorelbine in combination with epirubicin as first-line chemotherapy of patients with MBC. In all, 49 patients with MBC received, as first-line treatment, a combination regimen consisting of i.v. vinorelbine 25 mg m^−2^ plus epirubicin 90 mg m^−2^ given on day 1, and oral vinorelbine 60 mg m^−2^ on day 8 (or day 15 if neutrophils <1500 mm^−3^) every 3 weeks, in an open-label, multicentre phase II study. Treatment was to be repeated for a maximum of six cycles. The study population had a median age of 55 years, half of the patients had received prior adjuvant chemotherapy and 86% presented a visceral involvement. In all, 25 responses were documented and validated by an independent panel review, yielding response rates of 51% (95% CI: 36–66) in the 49 enrolled patients and 54.5% (95% CI: 39–70) in the 44 evaluable patients. Median durations of progression-free survival and survival were 8 and 20 months, respectively. Neutropenia was the main dose-limiting toxicity, but complications were uncommon, four patients having experienced febrile neutropenia and six having developed neutropenic infection. Other frequently reported adverse events included stomatitis, nausea and vomiting, which were rarely severe. No toxic death was reported. Among patients who received six cycles, global score of quality of life remained stable. This regimen alternating oral and i.v. vinorelbine in combination with epirubicin is effective and safe. Oral vinorelbine on day 8 offers greater convenience to the patient, and decreases the need for i.v. injection and reduces time spent in hospital. Therefore, oral vinorelbine is a convenient alternative to the i.v. form in combination regimens commonly used to treat MBC.

Breast cancer is the most common malignancy affecting women in the Western world. Over the past decade, the lifetime risk of developing breast cancer has been 12.2%. In Europe, the yearly incidence is approximately 80 cases per 100 000 women and approximately half of them will die of the disease. Therapy for metastatic breast cancer (MBC) has not improved significantly in recent years, remaining strictly palliative in nature and intent. While selected patients with advanced disease may have their survival prolonged by combination chemotherapy, no patients are cured. In this palliative setting, introduction of new regimens that can improve patient comfort and convenience is highly desirable.

Intravenous (i.v.) vinorelbine has been widely investigated in the treatment of MBC. Response rates of 35–50% have consistently been demonstrated for first-line single-agent vinorelbine (Fumoleau *et al*, 1993; Garcia-Conde *et al*, 1994; Romero *et al*, 1994; Twelves *et al*, 1994; Bruno *et al*, 1995; Weber *et al*, 1995; Terenziani *et al*, 1996). The good tolerance profile of i.v. vinorelbine has enabled its use in combination with other cytotoxic agents active against MBC. It has been safely and effectively combined with epirubicin in several noncomparative studies (Chadjaa *et al*, 1993; Ezzat *et al*, 1996; Baldini *et al*, 1998; Tabiadon *et al*, 1998; Nistico *et al* 1999; Vici *et al*, 2002). Recently, B Ejlertsen reported the results of a randomised phase III study comparing the combination of i.v. vinorelbine 25 mg m^−2^ on days 1 and 8 and epirubicin 90 mg m^−2^ on day 1 with single-agent epirubicin 90 mg m^−2^ on day 1, both regimens being given every 3 weeks (Ejlertsen *et al*, 2001). The combination of i.v. vinorelbine and epirubicin demonstrated a trend for higher rate of objective responses (50 *vs* 42%) and significantly longer duration of progression-free survival (10.1 *vs* 8.2 months). Leucopenia-related complications, stomatitis and peripheral neuropathy were more common for the combination regimen, but the incidences of cardiotoxicity, constipation and injection site reactions were similar in the two study arms. This phase III study established that addition of vinorelbine conferred a significant advantage over epirubicin used as a single agent in the first-line treatment of MBC.

Oncology is one of the few areas of medicine where most patients are treated i.v. rather than receiving oral drugs. Work from Liu and colleagues (Liu *et al*, 1997) has indicated that approximately 90% of cancer patients expressed a preference for oral *vs* i.v. chemotherapy, predominantly because of the convenience of administration outside a hospital setting or current concerns or previous problems with i.v. access. From a patient perspective, the availability of oral agents would make a significant contribution to patient's quality of life, provided that the efficacy and toxicity of these agents were comparable to that of their i.v. counterparts. Oral vinorelbine belongs to the new generation of oral drugs that achieve reliable blood exposure. Its bioavailability is about 40%, which indicates that 80 mg m^−2^ orally corresponds to 30 mg m^−2^ i.v. and 60 mg m^−2^ orally to 25 mg m^−2^ i.v. (Marty *et al*, 2001). Used as a single agent for the first-line treatment of MBC patients, oral vinorelbine was shown to be an effective and well-tolerated agent (Freyer *et al*, 2003; Trillet-Lenoir *et al*, 2004).

In two phase II studies, consistent response rates of 30% were reported. Also, median durations of progression-free survival and survival fall in the same range: 4.2 and 24 months in one trial and 4.6 and 21 months in the other trial. Similarly to i.v. vinorelbine, neutropenia was the main dose-limiting toxicity, but was rarely complicated: only 4% of patients enrolled in the two phase II studies experienced febrile neutropenia. No severe infection was reported. Nausea and vomiting were more frequently reported with oral vinorelbine in contrast to the usually low incidence seen with the i.v. form. However, they were generally of mild-to-moderate intensity. In subsequent studies of oral vinorelbine, a primary prophylaxis with oral 5-HT3 antagonist was used and was shown to easily control the occurrence of nausea and vomiting (Gridelli *et al*, 2003).

Oral vinorelbine is, therefore, a useful alternative to the i.v. form and deserves further clinical investigations in combination regimens. The present study was designed to evaluate vinorelbine, alternating i.v. on day 1 and oral on day 8, in combination with epirubicin infused on day 1 every 3 weeks in patients with MBC.

## MATERIALS AND METHODS

### Eligibility

Eligible patients fulfilled all the following criteria: progressive metastatic breast cancer; female aged ⩾18 and ⩽75 years; Karnofsky performance status ⩾70%; estimated life expectancy ⩾12 weeks; and who had adequate bone marrow, hepatic and renal functions (defined as neutrophils ⩾2.0 × 10^9^ l^−1^, platelets ⩾100 × 10^9^ l^−1^, haemoglobin ⩾10 g dl^−1^ or 6.2 mmol l^−1^, total bilirubin ⩽1.5 × upper limit of normal (ULN), AST and ALT ⩽2.5 × ULN, creatinine ⩽1.5 × ULN). Patients were required to have at least one bidimensionally measurable target lesion (documented by CT or MRI according to WHO criteria), measured within 21 days of inclusion in the study; physical examination, ultrasound and chest X-ray were not considered as objective tumour assessments. Prior therapy was permitted as follows: a minimum of 2 weeks had to have elapsed between surgery and inclusion in the study; patients might have had previous hormonal therapy as adjuvant treatment and/or treatment of metastatic disease provided that they had progressive disease at study entry; previous neoadjuvant and/or adjuvant chemotherapy that might have contained an anthracycline was allowed provided that an interval of at least 12 months had elapsed between the end of adjuvant chemotherapy and disease progression (for prior adjuvant anthracycline/anthracenedione containing regimen, a maximum prior dose of 180 mg m^−2^, 360 mg m^−2^ and 72 mg m^−2^ of doxorubicin, epirubicin or THP-adriamycin, and mitoxantrone was allowed, respectively). No delay between the end of adjuvant nonanthracycline/anthracenedione chemotherapy and study entry was required provided that the patient had fully recovered from toxic effects of prior adjuvant chemotherapy. Previous radiation therapy may have been given provided that 4 weeks had elapsed prior to study entry. However, the measurable disease had to be completely outside the radiation field.

Cardiac function should be normal as demonstrated by LVEF measured by radionuclide angiography (MUGA scan) or bidimensional echocardiography performed within 3 weeks to study entry.

The protocol was submitted to independent ethics committees. Their approvals were to be obtained prior to the start of the study. The study was conducted in accordance with the ethical principles set forth in the Declaration of Helsinki (Somerset West amendment) and in compliance with all applicable local regulations. Written informed consent was obtained from each participating patient prior to entry into the study.

### Treatment plan

The combination regimen consisted of i.v. vinorelbine 25 mg m^−2^ plus epirubicin 90 mg m^−2^ given on day 1, and oral vinorelbine 60 mg m^−2^ on day 8, every 3 weeks. Each patient received the study drugs for a maximum of six cycles unless disease progression or unacceptable toxicity. Treatment could be modified in the case of haematological or nonhaematological toxicity, but the duration of one cycle had not to exceed 5 weeks. Day 8 administration of oral vinorelbine could be delayed by only 1 week; if oral dosing could not be carried out on day 15, it had to be omitted.

In all centres involved in this clinical trial, the patient received the study treatment at the hospital. Dose delay and/or cancellation were depending on complete blood count results obtained within 24 h and checked by the physician prior to dosing.

### Treatment evaluation

Evaluation at study entry included physical examination, chest-ray completed by chest CT scan if lung metastases, liver ultrasound completed by abdominal CT scan if liver metastases and bone scintigraphy. All positive imaging procedures had to be repeated every two cycles and at the end of treatment. Thereafter, patients were followed every 3 months until death. WHO criteria were used to define response. Response rate was the primary efficacy variable. All registered patients were included in the efficacy analysis (intent-to-treat analysis). Patients evaluable for efficacy were defined as those who remained in the study until completion of the first evaluation (after first two cycles) as required by protocol and whose baseline lesions were all assessed with the same method of measurement throughout the study period. All responses were validated by an independent radiologist.

Progression-free survival was calculated from the registration date until the date of progression or death due to any cause. Survival was defined as the time elapsed from registration date until death or last contact.

Toxicity was evaluated by using the National Cancer Institute criteria (version 2.0). Cardiac monitoring during the study treatment included ECG before each cycle and assessment of LVEF, if clinically indicated.

Quality of life was evaluated by using the EORTC QLQ-C30 and QLQ-BR23 questionnaires.

### Statistical analysis

The primary study objective was to assess the response rate. Secondary objectives included safety evaluation, impact on quality of life and determination of the duration of response, progression-free survival and survival.

This study was an open-label, multicentre, noncomparative phase II trial. The one-sample multiple testing procedure of Fleming for phase II clinical trials was used. A minimum of 20 evaluable patients and a maximum of 40 evaluable patients depending on the response rate observed in the first 20 subjects were required. The procedure employed the standard single-stage test procedure at the last one of *k* prespecified testing, while both allowing for early termination (should extreme results be seen) and essentially preserving the size and power of the single-stage procedure. The reference responses rates, acceptable error probabilities and number of testings selected for this study were as follows: Po=40%, Pa=60%, *α*=5%, *β*=10%, *k*=2. This assumed that 40% was the minimum desirable response rate for an active combination therapy in this population. Under these conditions, the total sample size (*N*) was 40 evaluable patients and the first test was performed after 20 evaluable patients.

## RESULTS

### Patients characteristics

In all, 49 patients with MBC were included in the study between October 2000 and March 2002.

Patient and tumour characteristics are shown in Table 1. The study population was rather young with a median age of 55 years. A total of 28 patients (57%) had received prior adjuvant chemotherapy, which contained anthracycline or anthracenedione in 16 patients (57%). The majority presented visceral lesions, which involved the liver (51% of the patients) or the lung (37%).

### Treatment delivery

A total of 259 cycles were administered. The median number of cycles was 6 with a range 1–7. Of note, 82% of the patients remain under treatment until the fifth cycle and 74% complete the sixth cycle. Excessive toxicity and disease progression were responsible for the study discontinuation of four patients (8%) each. Other reasons included patient's refusal, investigator's decision for two patients (4%) each and nontoxic death for one patient.

Only few administrations were delayed, leading to high median relative dose intensities. Among the 49 treated patients, the median relative dose intensities for i.v. and oral vinorelbine were 95 and 85%, respectively, while for epirubicin, median RDI was 95%. Cycles were delayed for more than 3 days in 27 patients (55%) and for 45 cycles (21%). Administrations of oral vinorelbine on day 8 were delayed to day 15 for seven patients (14%) and for 10 cycles (4%) and cancelled for 18 patients (37%) and for 38 cycles (15%). Overall 20 patients had at least one day 8 either delayed or cancelled, while 29 patients (59%) received all oral administrations as per protocol. The principal reason for oral vinorelbine dose delay and cancellation was haematological toxicity.

### Efficacy

Among the 49 enrolled patients, 44 were evaluable for efficacy. The reasons for nonevaluability were major violation of eligibility criteria for three patients (one without bidimensionally measurable lesion, one with incomplete assessment of target lesions at baseline and one with severe ischaemic heart disease), premature discontinuation for one patient who refused further therapy after having received one cycle and lack of tumour assessment for the last patient.

A total of 25 responses (two complete and 23 partial) were reported, yielding an overall response rate of 51% (95% CI: 36–66) in intent-to-treat analysis (Table 2). Out of the three noneligible patients, one was assessed as a partial responder, one as no change and the last one was nonevaluable due to premature study discontinuation. The proportion of responders was similar in patients who had received prior anthracycline/anthracenedione containing adjuvant chemotherapy (eight of 16 patients: 50%) and in those who had not (17 of 33: 52%) In the 44 evaluable patients, the overall response rate was 54.5% (95% CI: 39–70). The median duration of response was 7.7 months (95% CI: 6.9–12.1). The median durations of progression-free survival and survival were 8 months (95% CI: 6.9–9.8) and 20 months (95% CI: 15.3–25.3), respectively.

### Toxicity

Toxcity is presented in Table 3. As expected from the toxicity profile of the study drugs, neutropenia was the main dose-limiting toxicity. Grade 3 and 4 neutropenia was seen in eight (16%) and 24 (49%) patients, respectively. Febrile neutropenia defined as grade 4 neutropenia concomitant with fever >38°C was reported in four patients (8%) and neutropenic infection defined as grade ⩾3 infection concomitant with grade ⩾3 neutropenia was seen in six patients (12%). In all instances, these complications resolved under antibiotic therapy.

Among the nonhaematological toxicities, gastrointestinal disorders were the most frequently reported. The proportions of patients who experienced nausea and vomiting were 86 and 59%, respectively, but only one and three patients complained of grade 3 or 4, respectively. Stomatitis occurred for 63% of patients and was scored as grade 3 for five patients (10%). Fatigue was also common (78% of patients).

Neurotoxicity was uncommon with nine patients (18%) having experienced mild-to-moderate neurosensory disorders and nine developing constipation, which was assessed as grade 3 in only one of them. Cardiotoxicity was minimal: one transient episode of arrhythmia was observed. Alopecia was almost universal (92% of patients). No toxic death occurred.

### Quality of life

In all, 36 patients completed at least one questionnaire at baseline and during treatment and 22 (45%) of them had completed regular quality of life assessments along the study until the fifth cycle (only one patient completed a questionnaire after the sixth and last administration). Results of the analysis of these two populations were similar but more meaningful in the second group of patients.

**Figure 1:**
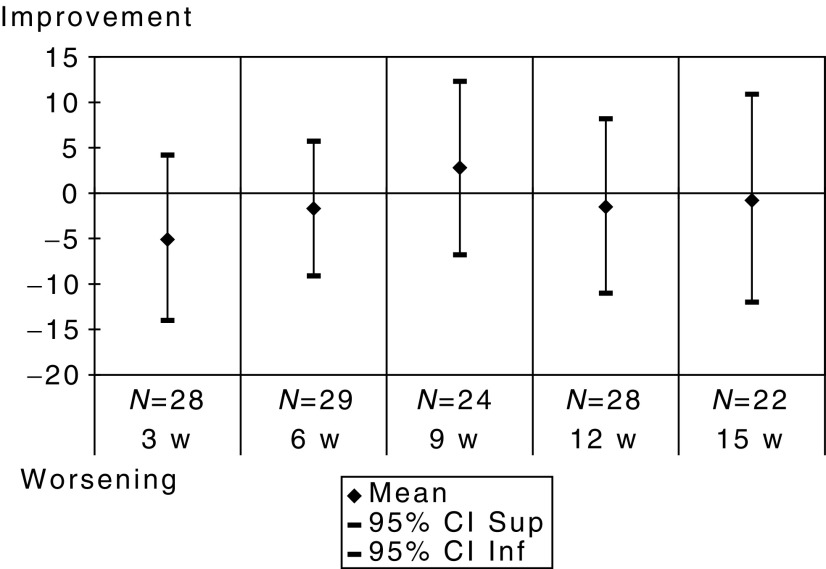
Mean differences of scores with baseline of the 22 patients who completed all QLQ-C30 questionnaires – global health status.

**Figure 2:**
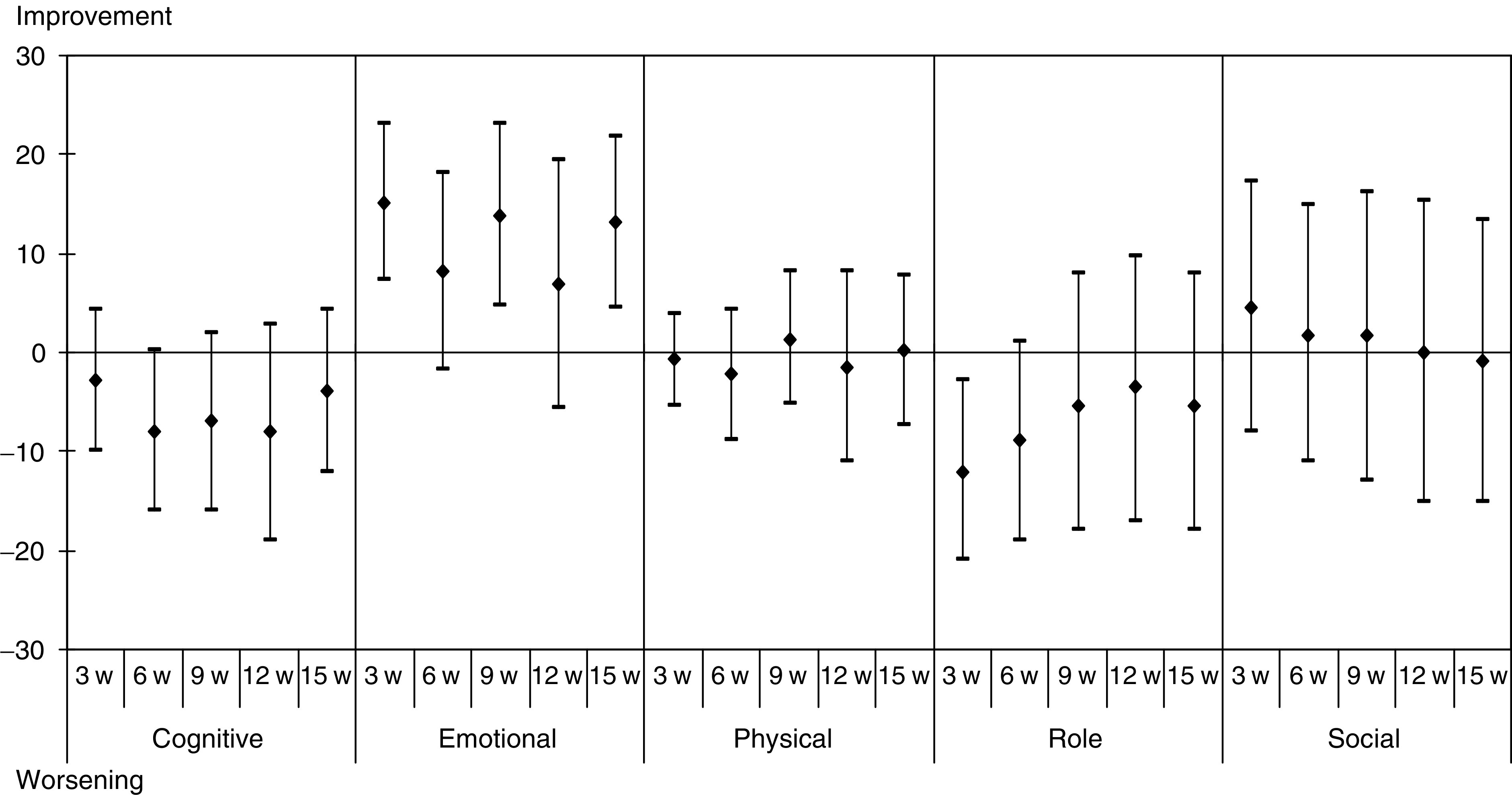
Mean differences of scores with baseline of the 21 patients who competed all QLQ-C30 questionnaires – functional scores.

**Figure 3:**
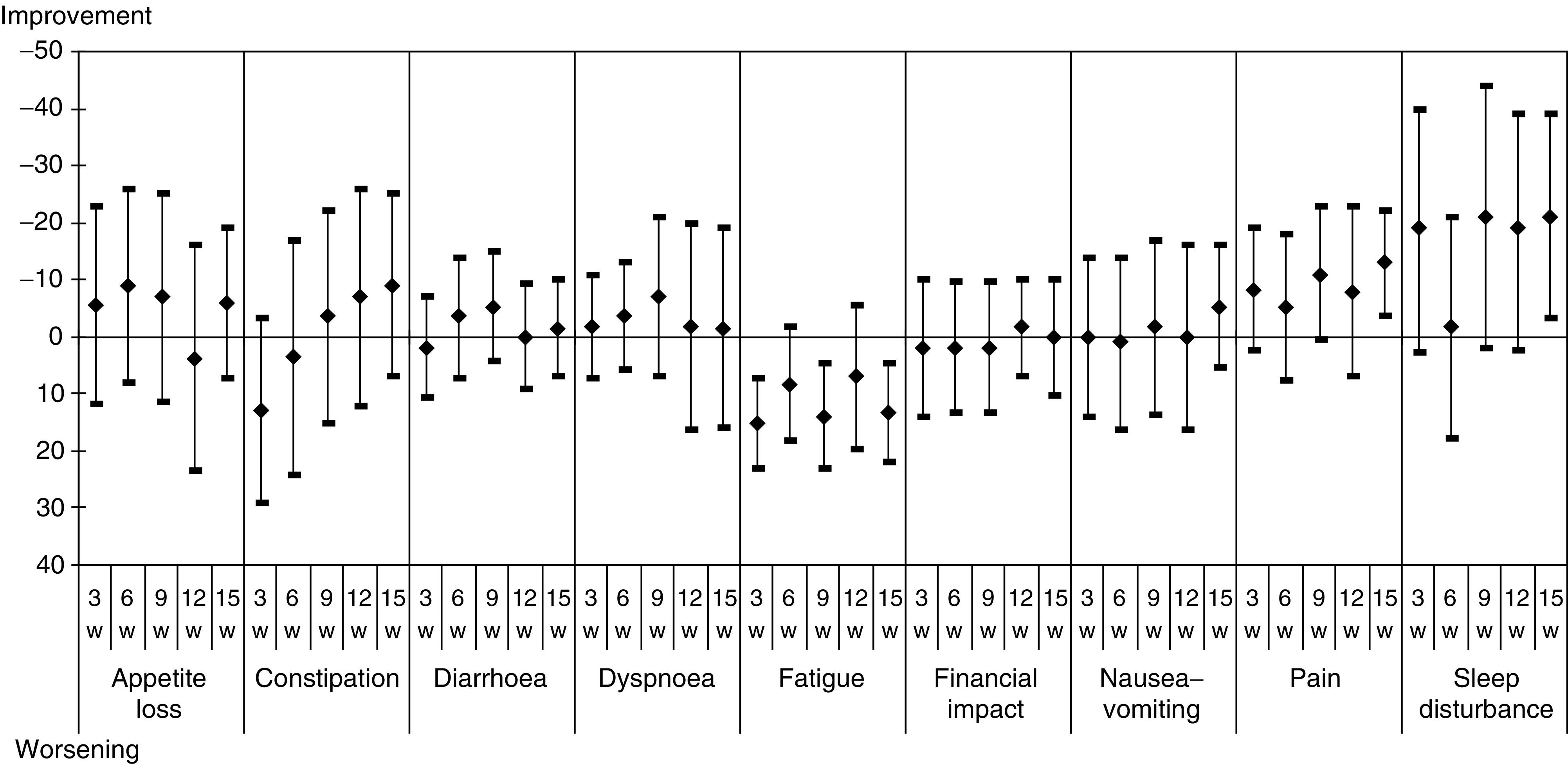
Mean differences of scores with baseline of the 22 patients who competed all QLQ-C30 questionnaires – symptoms scores.

The analysis of the QLQ-C30 questionnaire showed that patients did not feel a worsening of their global health status (Figure 1). Mean changes from baseline in functional and symptom scores are presented in Figures 2 and 3.

**Figure 4:**
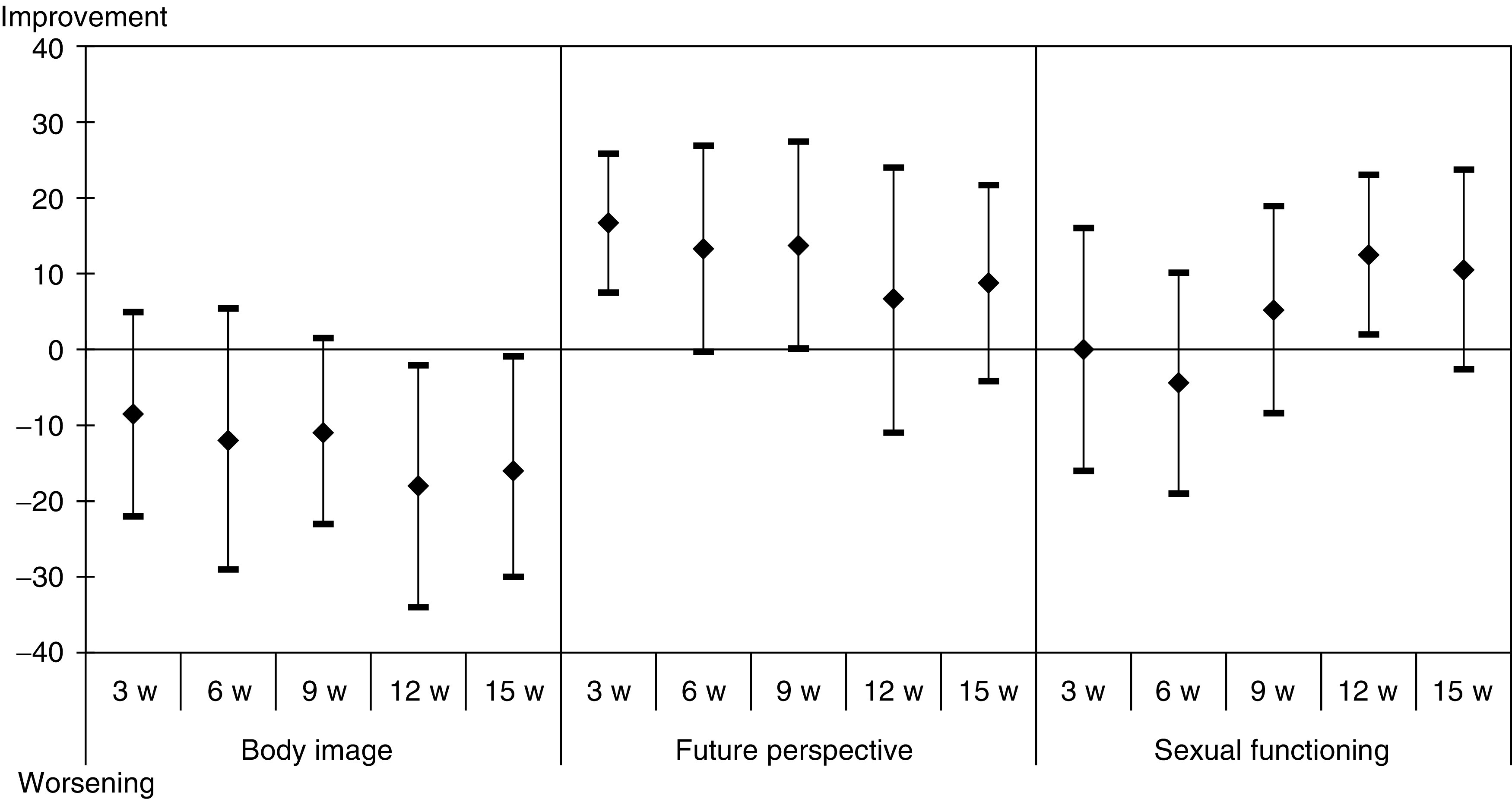
Mean differences of scores with baseline of the 21 patients who competed all QLQ-BR23 questionnaires – functional scores.

**Figure 5:**
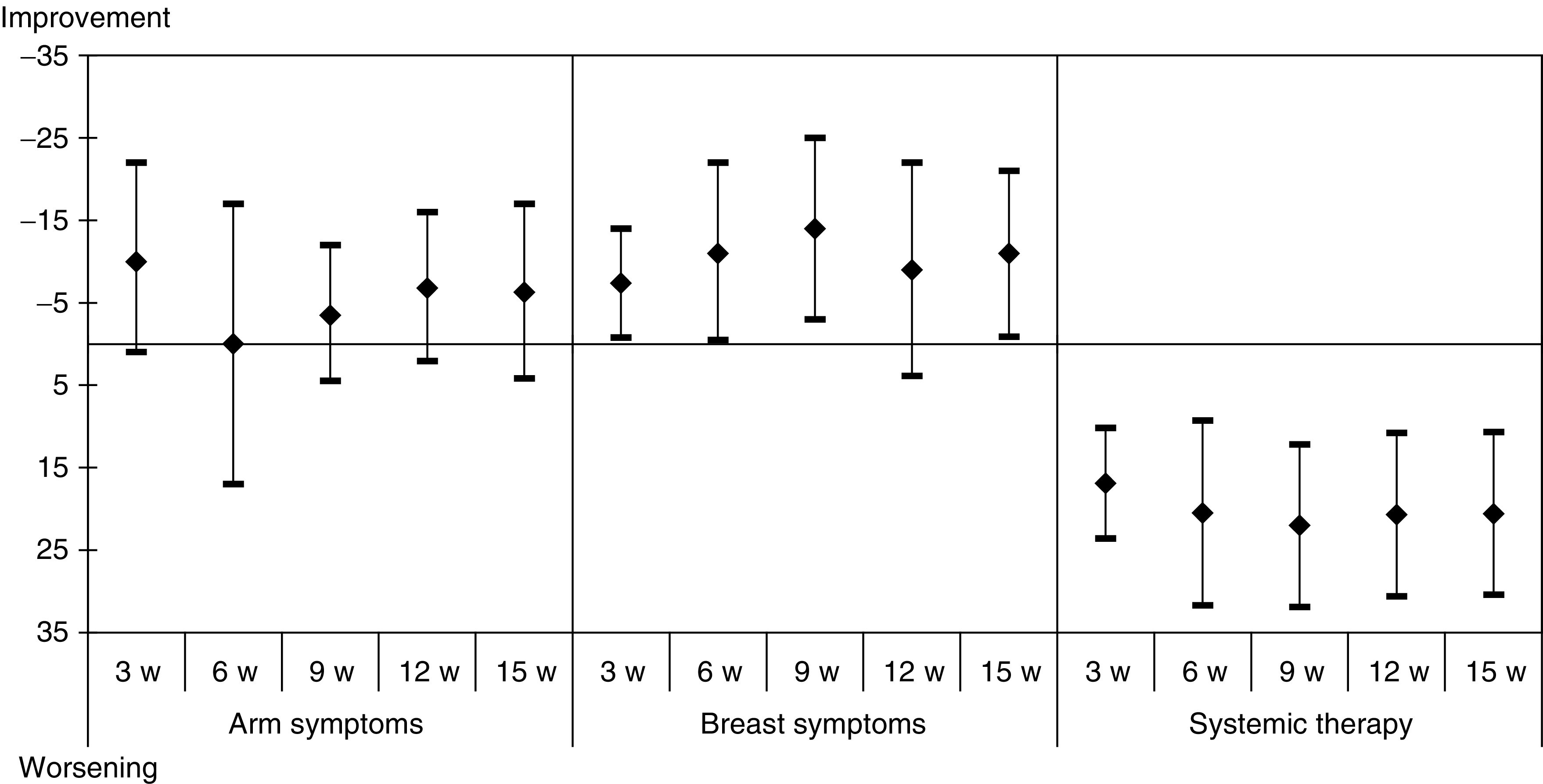
Mean differences of scores with baseline of the 21 patients who competed all QLQ-BR23 questionnaires – symptoms scores.

The cognitive and role functional scores worsened, while the emotional score improved. Regarding symptom scores, an improvement of appetite, pain and sleep was observed, while fatigue worsened Figures 4 and 5.

The BR23 questionnaire was completed by 21 patients (43%) at study entry and at all subsequent evaluations. Mean changes in the scores from baseline are presented in Figure 2. Body image and sexual functional scores worsened, but patients became less worried about their health in the future. Breast and arm symptoms improved, while systemic therapy score that gathered chemotherapy side effects worsened.

## DISCUSSION

Oral chemotherapy offers significant advantages over i.v. administration because of its greater convenience for the patient, its ease of administration and reduced need for hospitalisation. Patients with incurable cancer showed a clear preference for oral chemotherapy (Liu *et al*, 1997; Borner *et al*, 2002) provided that its efficacy is similar to the i.v. alternative. However, physicians have been reluctant to use oral cytotoxic drugs, in the past, because of important interpatient variations in drug disposition. With the new generation of oral cytotoxic drugs including oral fluoropyrimidines (De Mario *et al*, 1998) and oral vinorelbine (Marty *et al*, 2001), reliable blood exposure has been achieved. Therefore, these new drugs are now progressively replacing their i.v. counterpart.

In the palliative treatment of metastatic breast cancer, oral vinorelbine used as a single agent was shown to be an effective and well-tolerated treatment (Freyer *et al* 2003; Trillet-Lenoir *et al*, 2004). The next logical step has been to test oral vinorelbine in combination chemotherapy regimens, which have been shown to be efficient with i.v. vinorelbine.

For combination regimens, which contain a cytotoxic that is not orally available, a regimen using i.v. vinorelbine on the day the other cytotoxic is infused and oral vinorelbine for the rest of the cycle was investigated in an effort to improve patient convenience.

Clinical experience on the combination of i.v. vinorelbine and epirubicin in the first-line treatment of MBC is rather extensive based on several phase II studies (Chadjaa *et al*, 1993; Ezzat *et al*, 1996; Baldini *et al*, 1998; Tabiadon *et al*, 1998; Nistico *et al* 1999; Vici *et al*, 2002) and a phase III study conducted by the Danish Breast Cancer Group (DBCG) (Ejlertsen *et al*, 2001).

In phase II studies, i.v. vinorelbine was generally given at 25 mg m^−2^ on days 1 and 8 (Chadjaa *et al*, 1993; Ezzat *et al*, 1996; Baldini *et al*, 1998; Tabiadon *et al*, 1998) or on days 1 and 5 (Vici *et al*, 2002) of every 3-week cycles. Epirubicin was combined on day 1 of cycles at doses ranging from 60 to 100 mg m^−2^.

Vici *et al* chose to administer epirubicin 100 mg m^−2^ and i.v. vinorelbine on days 1 and 5 with G-CSF growth factor support every 3 weeks. Nitisco *et al* explored a dose-dense regimen where i.v. vinorelbine 25 mg m^−2^ and epirubicin 25 mg m^−2^ were infused weekly with growth factor support. Response rates ranged from 50 to 77%, the highest rate being achieved with the weekly dose-dense regimen.

The largest experience has come from the phase III study, which enrolled a total of 387 patients including 193 treated with i.v. vinorelbine 25 mg m^−2^ on days 1 and 8 and epirubicin 90 mg m^−2^ on day 1 every 3 weeks (Ejlertsen *et al*, 2004). This combination regimen achieved a response rate of 50% in the intent-to-treat analysis and 55% in the 175 evaluable patients. Median durations of progression-free survival and overall survival were 10.1 and 19.1 months, respectively. The most important adverse event was leucopenia, which was severe in 50% of patients. It was associated with fever in 20% of patients or severe infection in 11%. Stomatitis was frequent but severe in only 15% of patients. The incidences of peripheral neuropathy were 39%, but grade 3 or 4 events were rare. Only 5% of patients experienced grade 3 or 4 constipation. The addition of vinorelbine to epirubicin did not increase the risk of cardiotoxicity.

In the present study, a response rate of 51% was achieved in the intent-to-treat population and of 54.5% in the 44 evaluable patients. These results are consistent with prior experience obtained with a fully i.v. regimen. Similarly, median progression-free survival of 8 months and median overall survival of 20 months fall in the range expected from the results of the DBCG study.

As previously reported in the DBCG study, the main toxicities encountered in this phase II study included neutropenia and related events as febrile neutropenia (8% of patients) and neutropenic infection (12%), nausea/vomiting, stomatitis and fatigue. Neurotoxicity was uncommon and cardiotoxicity was minimal. No fatal event occurred during the study. This safety profile is consistent with the tolerance of the fully i.v. regimen.

The day 8 dosing of oral VRL was administered at hospital to check results of blood cells counts and ensure patient's compliance. Oral VRL administered at hospital already offers benefit to the patient as it decreases the time spent in the hospital and the incidence of local reaction or stress related to chemotherapy infusion. Benefit is also for the chemotherapy unit as oral VRL on day 8 reduces nursing time and costs.

In clinical practice, patient comfort would also be improved by day 8 oral administration at home provided that hospital team is trained to manage such patients by phone call and relationship with the general practioner.

In conclusion, this study indicated that partial substitution of i.v. vinorelbine by its oral form in combination with epirubicin seems as effective as the regimen using exclusively i.v. vinorelbine. In the future, home chemotherapy could be an effective and safe alternative to outpatient treatment, especially if a fully oral regimen combining oral vinorelbine with another orally available cytotoxic agent is developed.

## Figures and Tables

**Table 1:** Patient and tumour characteristics

	***N* (%)**
No. of patients	49
	
*Age (years)*	
Median	55
Range	[27–75]
	
Median disease-free interval (months)	34
	
*Menopausal status*	
Pre	33 (67)
Post	16 (33)
	
*Estrogen receptors*	
Positive	27 (55)
Negative	14 (29)
Unknown	8 (16)
	
*Prior therapy*	
Radiotherapy	32 (65)
Adjuvant chemotherapy	25 (51)
Adjuvant hormonotherapy	13 (27)
Hormonotherapy for MBC	17 (35)
	
*Karnofsky performance status*	
100%	19 (39)
90–80%	24 (49)
70%	6 (12)
	
Visceral involvement	42 (86)
	
*No. of organs involved*	
1	10 (20)
2	21 (43)
⩾3	18 (37)

**Table 2:** Overall response rate

	**ITT patients (*n*=49)**		**Evaluable patients (*n*=44)**	
**Overall response**	***N***	**(%)**	***N***	**(%)**
Complete response	2	(4.1)	2	(4.6)
Partial response	23	(46.9)	22	(50.0)
**Response rate (CR+PR)**	**25**	**(51.0)**	**24**	**(54.5)**
No change	17	(34.7)	16	(36.4)
Progressive disease	4	(8.2)	4	(9.1)
Nonevaluable	3	(6.1)	—	—

ITT=intent to treat; CR=complete response; PR=partial response.

**Table 3:** Toxicity by patient and by cycle NCI/CTC

	**By patient**			**By cycle**		
**Adverse events by NCI/CTC**	**Overall incidence *N* (%)**	**Grade 3 *N* (%)**	**Grade 4 *N* (%)**	**Overall incidence *N* (%)**	**Grade 3 *N* (%)**	**Grade 4 *N* (%)**
Neutropenia	43 (87.8)	8 (16.3)	24 (49.0)	130 (50.4)	27 (10.5)	48 (18.6)
Leucopenia	44 (89.8)	15 (30.6)	12 (24.5)	159 (61.6)	44 (17.1)	15 (5.8)
Anaemia	48 (98.0)	5 (10.2)	1 (2.0)	224 (86.8)	7 (2.7)	1 (0.4)
Thrombocytopenia	26 (53.1)	1 (2.0)	2 (4.1)	58 (22.5)	5 (1.9)	2 (0.8)
						
*Infection*						
Neutropenic inf.	6 (12.2)	6 (12.2)	—	6 (2.3)	6 (2.3)	—
Inf. without neutropenia	13 (26.5)	—	—	28 (10.8)	—	—
Inf. other	1 (2.0)	—	—	1 (0.4)	—	—
Catheter inf.	1 (2.0)	1 (2.0)	—	1 (0.4)	1 (0.4)	—
						
*Gastrointestinal*						
Nausea	42 (85.7)	1 (2.0)	—	124 (47.9)	1 (0.4)	—
Vomiting	29 (59.2)	2 (4.1)	1 (2.0)	56 (21.6)	2 (0.8)	1 (0.4)
Diarrhoea	26 (53.1)	1 (2.0)	—	53 (20.5)	1 (0.4)	—
Dysphagia	11 (22.5)	—	—	7 (2.7)	—	—
Anorexia	5 (10.2)	—	—	83 (32.1)	6 (2.3)	—
Stomatitis	31 (63.3)	5 (10.2)	—	18 (7.0)	1 (0.4)	—
Constipation	9 (18.4)	1 (2.0)	—	23 (8.9)	1 (0.4)	—
Dyspepsia	9 (18.4)	1 (2.0)	—	11 (4.3)	—	—
Mouth dryness	7 (14.3)	—	—	124 (47.9)	1 (0.4)	—
						
*Dermatology*						
Alopecia	45 (91.8)	—	—	NA	NA	NA
Injection site reaction	19 (38.8)	1 (2.0)	—	39 (15.1)	1 (0.4)	—
						
*Cardiovascular*						
Phlebitis	1 (2.0)	—	—	1 (0.4)	1 (0.4)	—
Arrhythmia	1 (2.0)	1 (2.0)	—	1 (0.4)	1 (0.4)	—
						
*Neurological*						
Dizziness	6 (12.2)	—	—	9 (3.5)	—	—
Neurosensory	9 (18.4)	—	—	21 (8.1)	—	—
Vertigo	5 (10.2)	—	—	9 (3.5)	—	—
						
*Constitutional*						
Fatigue	38 (77.6)	3 (6.1)	—	140 (54.1)	6 (2.3)	—
Fever in the absence of neutropenia	7 (14.3)	—	—	7 (2.7)	—	—
						
*Pain*						
Abdominal	10 (20.4)	—	—	16 (6.2)	—	—
Arthralgia	6 (12.2)	2 (4.1)	—	16 (6.2)	2 (0.8)	—
Headache	9 (18.4)	1 (2.0)	—	21 (8.1)	1 (0.4)	—
Myalgia	8 (16.3)	—	—	19 (7.3)	—	—
Pain other	6 (12.2)	—	—	13 (5.0)	—	—

NA=not applicable.

NCI/CTC =National Cancer Institute/Common Toxicity Criteria. In all, 258 cycles were evaluable for haematological toxicity.

In all, 259 cycles were evaluable for nonhaematological toxicity.
